# CREB Is Critically Implicated in Skin Mast Cell Degranulation Elicited via FcεRI and MRGPRX2

**DOI:** 10.3390/cells13201681

**Published:** 2024-10-11

**Authors:** Zhuoran Li, Jean Schneikert, Shiva Raj Tripathi, Manqiu Jin, Gürkan Bal, Torsten Zuberbier, Magda Babina

**Affiliations:** 1 Fraunhofer Institute for Translational Medicine and Pharmacology ITMP, Immunology and Allergology IA, 12203 Berlin, Germany; zhuoran.li@charite.de (Z.L.); jean.schneikert@charite.de (J.S.); shiva-raj.tripathi@charite.de (S.R.T.); manqiu.jin@charite.de (M.J.); guerkan.bal@charite.de (G.B.); torsten.zuberbier@charite.de (T.Z.); 2 Institute of Allergology, Charité—Universitätsmedizin Berlin, Freie Universität Berlin and Humboldt Universität zu Berlin, Hindenburgdamm 30, 12203 Berlin, Germany

**Keywords:** mast cell, CREB, FcεRI, MRGPRX2, degranulation, skin, flow cytometry, RTqPCR, RNA interference

## Abstract

Skin mast cells (MCs) mediate acute allergic reactions in the cutaneous environment and contribute to chronic dermatoses, including urticaria, and atopic or contact dermatitis. The cAMP response element binding protein (CREB), an evolutionarily well conserved transcription factor (TF) with over 4,000 binding sites in the genome, was recently found to form a feedforward loop with KIT, maintaining MC survival. The most selective MC function is degranulation with its acute release of prestored mediators. Herein, we asked whether CREB contributes to the expression and function of the degranulation-competent receptors FcεRI and MRGPRX2. Interference with CREB by pharmacological inhibition (CREBi, 666-15) or RNA interference only slightly affected the expression of these receptors, while KIT was strongly attenuated. Interestingly, MRGPRX2 surface expression moderately increased following CREB-knockdown, whereas MRGPRX2-dependent exocytosis simultaneously decreased. FcεRI expression and function were regulated consistently, although the effect was stronger at the functional level. Preformed MC mediators (tryptase, histamine, *β*-hexosaminidase) remained comparable following CREB attenuation, suggesting that granule synthesis did not rely on CREB function. Collectively, in contrast to KIT, FcεRI and MRGPRX2 moderately depend on unperturbed CREB function. Nevertheless, CREB is required to maintain MC releasability irrespective of stimulus, insinuating that CREB may operate by safeguarding the degranulation machinery. To our knowledge, CREB is the first factor identified to regulate MRGPRX2 expression and function in opposite direction. Overall, the ancient TF is an indispensable component of skin MCs, orchestrating not only survival and proliferation but also their secretory competence.

## 1. Introduction

Mast cells (MCs) are critical effector cells in IgE-dependent type-I-hypersensitivity reactions, key events in urticaria, allergic rhinoconjunctivitis, allergic asthma, food allergy and anaphylaxis [1,2,3].

In the skin, where MC density is highest in the steady-state [4], MCs are overabundant and/or hyperactive and contribute to chronic diseases like atopic and contact dermatitis, mast cell activation syndrome, psoriasis, rosacea and other conditions [5,6,7]. Furthermore, inflammatory circuits initiated by the MC-neuronal crosstalk underly the sensation of pain and especially itch through operating units with sensory neurons [8,9,10,11,12]. As the receptor for various neuropeptides, MRGPRX2 (Mas-related G protein coupled receptor X2) is believed to chiefly mediate the latter responses [13,14,15,16,17,18,19,20,21,22,23].

While MRGPRX2 and FcεRI organize MC activation, KIT is the major receptor tyrosine kinase of the lineage. Together with its ligand SCF, KIT orchestrates differentiation from precursor cells, and regulates the survival, proliferation, and function of fully mature subsets [24,25,26,27,28]. SCF stimulation of skin MCs induces drastic changes in the proteome-wide phosphoproteome with ≈5400 out of ≈10,500 phosphosites being affected [29]. A substrate implicated in this pathway is CREB, which experiences robust phosphorylation in SCF-stimulated skin MCs [30].

CREB is an interesting transcription factor (TF) from several standpoints. It is one of the oldest and evolutionarily best-conserved TFs with over 4000 binding sites in the human genome [31]. This fits its involvement in a wide range of biological functions. Though largely ubiquitous, CREB expression levels are nevertheless regulated and particularly abundant in granulocytes, MCs, some other leukocytes, and the brain [32,33]. As a stimulus-inducible TF, CREB is constitutively present in the nucleus but requires posttranslational modification (especially phosphorylation at Ser-133) to drive transactivation by recruiting coactivators to the transcriptional machinery [34,35,36].

Since CREB function in MCs had received fairly little attention, limited mainly to its fate following activation [37,38,39,40], while regulating survival in other lineages [34,41,42,43,44], we recently queried whether skin MC maintenance requires an unperturbed CREB system. Indeed, by engaging in a circular relationship with KIT, CREB was found to be an indispensable component of skin MCs, whereby its absence abolished the skin MC compartment [45]. In particular, long-term interference with CREB resulted in nearly complete elimination, while a more nuanced and less drastic phenotype was observed following CREB suppression for a few days [45].

Here, we asked whether CREB is involved in the expression and function of degranulation-competent receptors. We report that short-term perturbation of CREB by a selective inhibitor or RNA interference (RNAi) modestly impacts MRGPRX2 or FcεRI expression in a positive (MRGPRX2) or negative (FcεRI) fashion, while potently suppressing MC degranulation independently of the eliciting route. This suggests that CREB may be involved in the maintenance of the degranulation machinery.

Our findings emphasize the factor’s relevance in skin MCs by highlighting that it may not only contribute to MC hyperplasia (e.g., in the context of mastocytosis) but also initiate or maintain inflammatory dermatoses through contribution to the complex program preceding degranulation [46,47].

## 2. Materials and Methods

### 2.1. Cells and Treatments

MCs were isolated from human foreskin tissue as described [48]. Each mast cell preparation/culture originated from several (2–15) donors to achieve sufficient cell numbers, as routinely performed in our lab [49,50,51,52]. The skin was obtained from circumcisions, with written, informed consent of the patients or legal guardians and approval by the university ethics committee (protocol code EA1/204/10, 9 March 2018). The experiments were conducted according to the Declaration of Helsinki Principles. Briefly, the skin was cut into strips and treated with dispase (26.5 mL per preparation, activity: 3.8 U/mL; Boehringer-Mannheim, Mannheim, Germany) at 4° C overnight. The epidermis was removed, and the dermis finely chopped, and digested with 2.29 mg/mL collagenase (activity: 255 U/mg; Worthington, Lakewood, NJ, USA), 0.75 mg/mL hyaluronidase (activity: 1000 U/mg; Sigma, Deisenhofen, Germany), and DNase I at 10 µg/mL (Roche, Basel, Switzerland). Cells were filtered stepwise from the resulting suspension (100 and 40 µm strainers, Fisher Scientific, Berlin, Germany). MC purification was achieved by anti-human c-Kit microbeads (#130-091-332) and the Auto-MACS separation device (both from Miltenyi-Biotec, Bergisch Gladbach, Germany), giving rise to 98-100% pure preparations (by acidic toluidine blue staining, 0.1% in 0.5 N HCl (Fisher Scientific, Berlin, Germany), as described [53,54]. In selected experiments, MCs were isolated from adult female skin obtained from breast reduction surgeries. Here, each preparation was from a single donor.

Purified skin MCs from individual preparations were cultured in Basal Iscove’s medium with 10% FCS (Biochrom, Berlin, Germany) in the presence of SCF (100 ng/mL), and IL-4 (20 ng/mL), freshly provided twice weekly when cultures were re-adjusted to 5 × 10^5^ /mL. MCs were automatically counted by CASY-TTC (Innovatis/Casy Technology, Reutlingen, Germany) [52,55].

For inhibition studies, cells were pre-incubated with 666-15 (CREB inhibitor; 5 µM; from Merck Chemicals, Darmstadt, Germany), or imatinib-mesylate (Gleevec, KIT inhibitor; 10 µM, from Biozol Diagnostica, Eching, Germany), or both combined for 30 min. Stimulation by SCF was at 100 ng/mL. It was reported that 666-15 inhibited the interaction between CREB and its co-activators, CREB binding protein and p300, and its potency and selectivity were shown in previous literature [56,57,58]. Cells were harvested after the times given in the legends and/or Results.

### 2.2. Accell^®^ Mediated RNA Interference

A well-established and efficient siRNA method for skin MCs was utilized [29,50,59,60]. In brief, skin MCs were transfected by CREB-targeting siRNA (E-003619-00-0050, Dharmacon, Lafayette, CO, USA) or control siRNAs ([30] (each at 1 µM) for 2 d in Accell^®^ medium (Dharmacon, Lafayette, CO, USA) (supplemented with 100 ng/mL SCF, and Non-Essential Amino Acids and L-Glutamine, both from Carl Roth, Karlsruhe, Germany).

### 2.3. Flow Cytometry

MCs were blocked with human AB serum (Biotest, Dreieich, Germany) for 15 min at 4 °C and then stained with either a specific anti-CD117 (Miltenyi-Biotec #130-111-593, Bergisch Gladbach, Germany) antibody, an anti-FcεRI-FITC (eBiosciene #11-5899-42, Fisher Scientific, Berlin, Germany) antibody or an anti-MRGPRX2-APC antibody (Biolegends, #359006, Amsterdam, The Netherlands) for 30 min at 4 °C. Corresponding isotype controls were used in each experiment. After incubation, cells were washed in phosphate-buffered saline (PBS) and resuspended in fluorescence activated cell sorting (FACS) buffer consisting of 2% fetal bovine serum in PBS. The cells were immediately processed in a Sony ID7000™ Spectral Cell Analyzer (Berlin, Germany) and gated on the population of identifiable, healthy cells in the forward scatter/side scatter plot, excluding debris and evidently dead cells. The data were analyzed with the FlowJo V10 analysis software (FlowJo LLC., Ashland, OR, USA).

### 2.4. Reverse Transcription-Quantitative PCR (RT-qPCR)

RNA was isolated using the NucleoSpin RNA kit from Macherey-Nagel (Düren, Germany) following the manufacturer’s instructions. cDNA synthesis (reverse transcription kit from Fisher Scientific) and RT-qPCR were performed using optimized conditions as described elsewhere [48] using materials from Roche (Roche Diagnostics, Mannheim, Germany). The primer pairs are summarized in Table 1. They were synthesized by TibMolBiol, Berlin, Germany. The 2^−ΔΔCT^ method was used to quantify the relative expression levels of the target genes to three reference genes (appearing at the end of Table 1).

### 2.5. β-Hexosaminidase Release Assay

Detection of MC degranulation by *β*-hexosaminidase quantification was performed as described [49,50]. Briefly, cells were treated with vehicle (spontaneous release), or challenged with codeine-phosphate at 100 μg/mL (solution prepared by the Charité pharmacy at 0.9% in water), substance P (SP) at 30 μM (Bachem, Budendorf, Switzerland) or anti-FcεRIα-Ab (clone AER-37, eBioscience, San Diego, CA, USA) at 0.1 μg/mL for 60 min in PAG-CM buffer (Piperazine-N,N-bis[2-ethanesulfonic acid]-Albumin-Glucose buffer containing 3 mM CaCl_2_ and 1.5 mM MgCl_2_, pH 7.4). Supernatants (SNs) were collected, and the pelleted MCs were rapidly frozen with 100 μL H_2_O at −80 °C. After thawing, 50 μL of SNs and lysates were incubated with 4-methyl umbelliferyl-N-acetyl-beta-D-glucosaminide (Sigma-Aldrich, Munich, Germany) solution at 5 μM in citrate buffer (pH 4.5) of the same volume and incubated for 60 min at 37 °C. Then, 100 mM sodium carbonate buffer (pH 10.7) was added to stop the reaction. Fluorescence intensity was determined at excitation at 355 nm and emission wavelength of 460 nm. Percent *β*-hexosaminidase release = [fluorescence intensity SN/(fluorescence intensity SN + fluorescence intensity lysate)] × 100. Net release was calculated by subtracting spontaneous release.

### 2.6. Quantification of Tryptase

Tryptase activity was measured according to an established protocol [61,62]. Then, 50,000 skin mast MCs were lysed in 100 µL of distilled water and 50 µL samples thereof were analyzed in 3–4 dilutions. Enzyme activity was determined by monitoring the cleavage of the peptide N-CBZ-Gly-Pro-Arg-pNA (Sigma-Aldrich, Munich, Germany) at 0.5 mg/mL. To 50 µL of each sample, 150 µL of sample buffer (150 mM Tris pH 7.6, 300 mM KCl, 50 µg/mL heparin) was added. To eliminate confounding enzyme activities, alpha-1 antitrypsin at a final concentration of 1 mg/mL served to suppress trypsin-like proteases. The changes in optical density per minute, caused by the cleavage of the substrate, were monitored, and recorded by measuring absorbance at 405 nm every 2 min on the VICTOR X5 2030 Multilabel HTS Microplate Reader (Perkin Elmer, Berlin, Germany).

### 2.7. Quantification of Histamine

The HTRF Histamine Dynamic kit (Revvity, Hamburg, Germany) was used according to the manufacturer’s instructions. As such, 1000 MCs were pelleted and lysed in 100 µL of distilled water. Different dilutions thereof were used for the assay. Optical densities were recorded on the VICTOR X5 2030 Multilabel HTS Microplate Reader, as above.

### 2.8. CD107a Exteriorization

CD107a exteriorization was performed as described previously [59,63]. In brief, MCs were stimulated by FcεRI-aggregation (for 15 min), or SP, or codeine (at 100 µg/mL) for 8 min, or no stimulus (control). The reaction was stopped by ice-cold 4% paraformaldehyde for 15 min. After washing, the cells were incubated with 10 µL of anti-human CD107a-FITC antibody (LAMP-1) (BD Pharmingen, Catalog number 555800, San Diego, CA, USA) together with 10 µL of human AB-serum for 30 min at 4 °C, then washed. CD107a expression was detected by flow cytometry as above.

### 2.9. Statistics

Statistical analyses were carried out using PRISM 8.0 (GraphPad Software, La Jolla, CA, USA). Comparisons between two groups were performed using the paired Student’s *t*-test. For comparisons across more than two groups, an RM one-way ANOVA with Dunnett’s multiple comparisons test was used. A one-sample *t*-test (normal distribution) was applied to assess the significance of normalized values. A *p* value of less than 0.05 was considered statistically significant.

## 3. Results

### 3.1. CREB Is Modestly Implicated in MRGPRX2 and FcεRI Expression in Skin MCs

**Figure 1:**
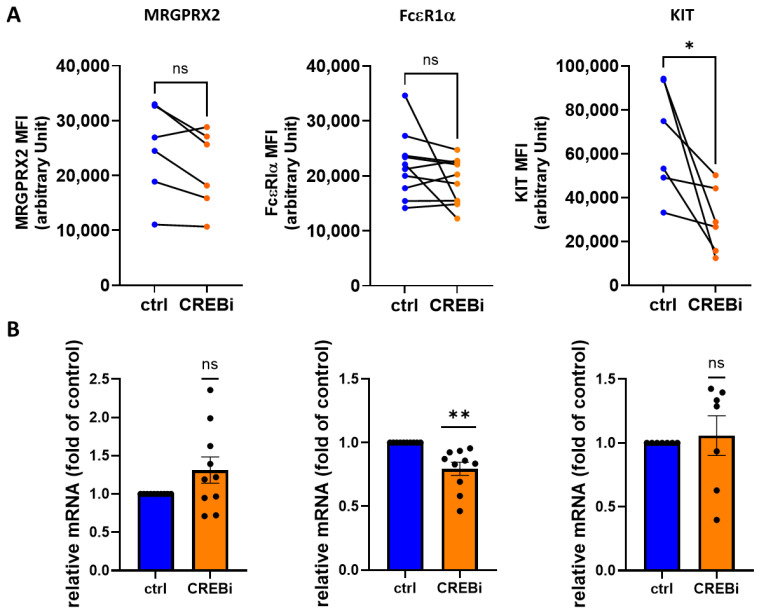
Pharmacological inhibition of CREB results in modest changes in MRGPRX2 or FcεRIα expression. Skin MCs were treated for 2 d with either the 666-15 inhibitor (CREBi) or vehicle (ctrl) and harvested for either flow cytometry analysis using specific antibodies to MRGPRX2, FcεRIα and KIT as indicated (**A**) or RT-qPCR (**B**). MFI, mean fluorescence intensity. In (**A**), results are given connecting dots of the same experiment. In (**B**), results (normalized to housekeeping genes as described in Methods) are expressed relative to the control set as 1 and given as mean ± SEM and individual dots. *, *p* < 0.05, **, *p* < 0.01, ns, not significant. (**A**) paired *t*-test, (**B**) one sample *t*-test or Wilcoxon test.

We recently reported that the major receptor tyrosine kinase of the MC lineage, i.e., KIT depends on unperturbed CREB activity in skin MCs [45]. Herein, we analyzed whether the TF molds the MC phenotype and function more broadly. MRGPRX2 and FcεRI are the major systems efficiently eliciting exocytosis of preformed mediators in skin MCs [49,52,59,64]. Using the selective CREB inhibitor 666-15, we found no major influence on MRGPRX2 or FcεRI surface expression (Figure 1A). As expected, KIT expression was potently reduced on CREBi treatment, making KIT the most susceptible of the studied receptors. At the mRNA level, no effect was noted for the MRGPRX2 or KIT transcript (Figure 1B), confirming that downregulation of KIT protein was caused by a post-transcriptional mechanism, as reported [45]. FcεRIα mRNA was modestly reduced to ≈80% of control (Figure 1B). In addition, the inhibitor had little effect on the expression of its own target, suggesting absence of major feedback or feedforward loops (Figure S2A). We also confirmed that CREB expression was comparable between female, adult and male, juvenile skin MCs in accordance with a previous report [65] (Figure S1).

**Figure 2:**
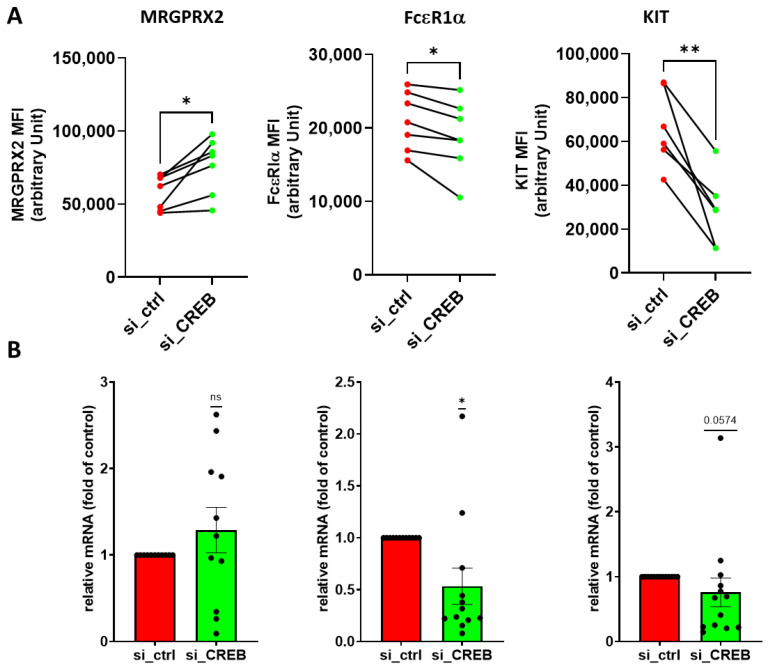
CREB-selective RNA interference results in a slight increase in MRGPRX2, and reduction of FcεRI expression. Skin MCs were transfected with either a control small interfering RNA (si_ctrl) or an siRNA specific to CREB (si_CREB) and harvested 2 d later for either flow cytometric analysis with specific antibodies to MRGPRX2, FcεRIα and KIT (**A**) or RT-qPCR (**B**). MFI, mean fluorescence intensity. In (**A**), results are given connecting dots of the same experiment. In (**B**), results (normalized to housekeeping genes as described in Methods) are expressed relative to the control set as 1 and given as mean ± SEM and individual dots. *, *p* < 0.05, **, *p* < 0.01, ns, not significant. (**A**) paired *t*-test, (**B**) one sample *t*-test or Wilcoxon test.

To ascertain that the above effects were mediated by CREB, we employed our established RNA interference strategy, which gives rise to a reduction of >50% [30,45], confirmed in the current study (Figure S2). This most selective strategy led to a slight increase in MRGPRX2 at the cell surface (Figure 2A) without significant modification of its transcript (Figure 2B), while simultaneously dampening FcεRI protein and mRNA (Figure 2). As expected, CREB suppression gave rise to robust reduction of KIT protein, while KIT mRNA was only weakly downregulated, confirming previous results [45]. We conclude that among lineage-specifying entities, KIT is the receptor whose expression most strongly depends on unperturbed CREB function.

### 3.2. CREB Contributes to SCF-Triggered Upregulation of FcεRI

**Figure 3:**
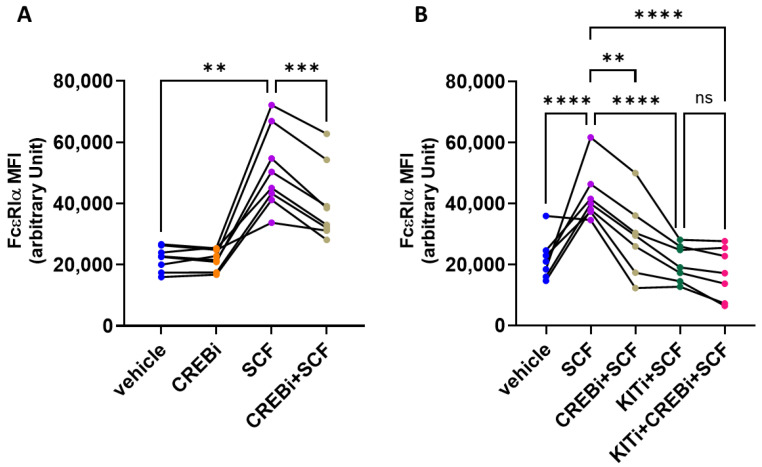
CREB is required for SCF mediated FcεRI upregulation. Skin MCs were pretreated with the vehicle control or the CREB inhibitor 666-15 (CREBi), either alone (**A**) or in combination with the KIT inhibitor imatinib-mesylate (KITi) versus KITi alone (**B**). Cells were stimulated 15 min later with SCF, where indicated. After 2 d, cells were harvested and submitted to flow cytometric analysis using an anti-FcεRIα antibody. MFI, mean fluorescence intensity. Each graph represents an individual experiment. **, *p* < 0.01, ***, *p* < 0.001, ****, *p* < 0.0001, using RM one-way ANOVA; ns, not significant.

FcεRIα expression and function are positively influenced by the SCF/KIT axis in skin MCs [52]. While in accordance with Figure 1, FcεRI expression was only slightly reduced following CREB inhibition, CREBi interfered with its upregulation by SCF (Figure 3A). We speculated that the latter stems from suppression of the SCF/KIT axis [45] and is therefore a consequence of CREB’s impact on KIT in the first place. The KIT kinase inhibitor imatinib mesylate (KITi) was used to corroborate this assumption. While KITi interfered with SCF, and completely abrogated FcεRI upregulation (green versus purple in Figure 3B), combining the two inhibitors had hardly any additional effect compared to KITi alone (magenta versus green in Figure 3B). Therefore, while CREB modestly contributes to FcεRI expression, it is required for SCF-triggered augmentation of FcεRI, and this occurs to a significant part through its influence on the SCF/KIT axis.

### 3.3. CREB Maintains the Secretory Competence of Skin MCs

**Figure 4:**
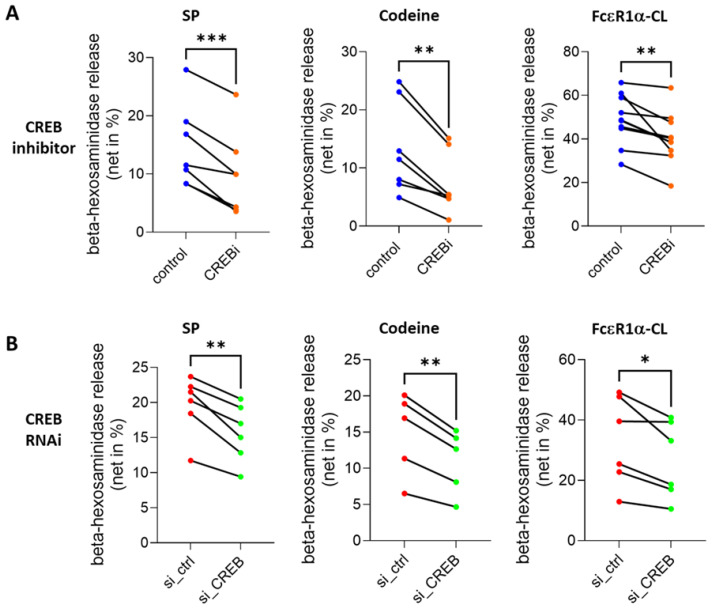
Unperturbed CREB activity is required for MRGPRX2- and FcεRI-elicited skin MC degranulation. Skin MCs were either pretreated with the CREB inhibitor 666-15 (CREBi) or vehicle (control) for 2 d (**A**) or transfected with control (si_ctrl) or CREB-targeting siRNA (si_CREB) for 2 d (**B**). Cell degranulation was induced with substance P, codeine or an anti-FcεRIα antibody. *β*-hexosaminidase activity was measured and is given as net release. *, *p* < 0.05, **, *p* < 0.01, ***, *p* < 0.001, using the paired *t*-test. CL, crosslinking.

Exocytosis is a complex process, orchestrated by a finely tuned interplay between hundreds of participants required for migration, tethering, docking, priming, and finally fusion of the secretory granules with the plasma membrane [46,47,66]. While expression of MRGPRX2 and FcεRI were only slightly affected by CREB perturbation (and if so, in opposite directions), we asked whether CREB-regulated processes may have a critical role in degranulation as such. In fact, pretreatment with CREBi for 2 d led to hyporesponsiveness to Substance P or codeine, two major ligands of the MRGPRX2 receptor (Figure 4A, left and center). A similar effect was noted for the FcεRI dependent route though the decrease in granule discharge was somewhat less steep than in the MRGPRX2 pathway (Figure 4A, right). The effects observed with the inhibitor were fully reproduced with our RNAi strategy (Figure 4B), highlighting the fact that CREB is vital to maintaining MC degranulability. To confirm the results of *β*-hexosaminidase release by an independent method, we measured the upregulation of CD107a, an activation marker externalized to the surface of on skin MCs upon MRGPRX2 ligand binding or FcεRI aggregation [59,67]. While pretreatment with CREBi nearly abolished CD107a upregulation stimulated by SP, codeine or FcεRI-crosslinking (Figure S3A), CREB-RNAi likewise diminished CD107a exteriorization induced by the three stimuli (Figure S3B). This confirmed that the maintenance of skin MC degranulability requires the continuous action of CREB.

**Figure 5:**
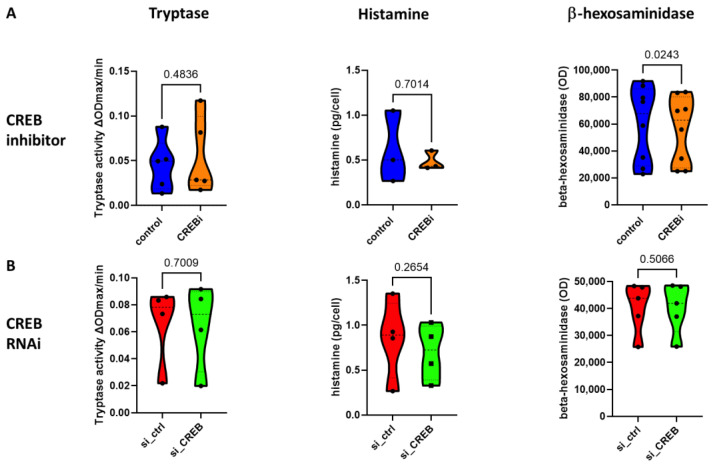
CREB has a minimal effect on the abundance of preformed mediators in skin MCs. Skin MCs were either pretreated with the CREB inhibitor 666-15 (CREBi) or vehicle (control) for 2 d (**A**) or transfected with control (si_ctrl) or CREB-targeting siRNA (si_CREB) for 2 d (**B**). The quantities of cell-contained tryptase, histamine, and *β*-hexosaminidase were determined in quiescent cells by the respective assays, as given in Methods. The data are depicted as violin plots with the *p*-values given above (paired *t*-test).

We also queried whether CREB influences the abundance of preformed MC mediators, by measuring tryptase, histamine and *β*-hexosaminidase following CREB manipulation. The quantity of granule-contained mediators remained comparable for both CREBi (Figure 5A) and CREB-RNAi (Figure 5B), however. This was found for tryptase, histamine and *β*-hexosaminidase alike, suggesting that granule architecture was largely maintained when CREB was not (fully) functional. This result also underlines the fact that CREB does not control the ratios of prefabricated mediators, since no shifts in favor of selected entities could be observed following CREB manipulation.

## 4. Discussion

Skin MCs take center stage in the orchestration of immune responses to invading pathogens, but they are also the root of acute hypersensitivity reactions and multiple dermatoses [3,5,6,68,69,70,71]. The most selective MC function is degranulation with its release of highly active preformed mediators, several of which unique to MCs [72]. In the skin, signs and symptoms that directly reflect preceding MC activation can be manifested as wheals (edema), flares, angioedema, and/or pruritus. In addition, skin MCs are major effector cells in anaphylaxis, the most severe clinical presentation of an acute allergic reaction, of which IgE-dependent and IgE-independent forms exist, the latter encompassing MRGPRX2 activation by a broad spectrum of drugs [15,73].

Understanding the process of MC exocytosis is therefore of great clinical significance in MC-dependent or -assisted skin diseases such as urticaria, angioedema, atopic and contact dermatitis, rosacea, prurigo, and psoriasis. Having recently reported that CREB has a nonredundant role in skin MC preservation, we asked herein whether its activity is also required for safeguarding the cells’ releasability. Indeed, both strategies employed (i.e., CREBi and CREB-RNAi) substantially reduced the secretory competence of skin MCs.

Of note, MRGPRX2-elicited degranulation was at least as strongly affected as the process triggered via FcεRI. Since (a slight) downregulation of the respective receptors was only found in the case of FcεRI, while MRGPRX2 showed the opposite trend, this result highlights the fact that CREB’s impact unlikely proceeds via modulation of receptor expression in the first place. It rather insinuates CREB’s role in the regulation of post-receptor events, possibly through transcriptional regulation of components making up the granule transport and exocytosis machinery [46,47,66]. This may explain why secretory competence downstream of MRGPRX2 was affected to the same or even greater extent than that downstream of FcεRI, even though MRGPRX2 was negatively regulated by CREB, whereby CREB-selective RNAi led to a moderate increase in its expression. The late signaling events of degranulation are highly complex and still less well investigated than the early steps that occur shortly after receptor stimulation. Notwithstanding, a large set of proteins is involved in the traffic, priming, tethering and docking of secretory granules, including SNARE (soluble N-ethylmale-imide-sensitive factor-attachment protein receptors) proteins, Rab, Munc13 and Munc18 family members, and Ca^2+^ sensors like synaptotagmins [47]. Regarding the large spectrum of Rab components, some have recently been described as coordinately regulating degranulation by different stimuli, including FcεRI and MRGPRX2 (e.g., RAB7, RAB12) [74]. It remains to be determined whether these genes form part of the CREB-regulated transcriptome, but if they do and their levels are decisive in dictating the magnitude of degranulation, it could provide a plausible explanation for the current findings. In support of this assumption, the nearly perfect correlation between FcεRI- and Ca++ ionophore elicited secretion in skin MCs [75] likewise suggests the existence of rate-limiting components downstream of receptor-proximal events. In further support, CREB is a master regulator of neurogenesis, and its target genes are implicated in neurotransmission [76], while neurotransmitter release and MC degranulation show many overlaps [77]. The overall dependence of skin MCs on CREB further highlights this intricate relationship between these otherwise unrelated lineages.

While this study used male MCs, we know from a genome-wide screen that male foreskin-derived and female breast skin-derived MCs show comparable expression of CREB1 (67, 64 tpm in the former versus 73 and 71 tpm in the latter) [65]. We could confirm this pattern herein by RT-qPCR (Figure S1). Previous data have also indicated that, although foreskin-derived and breast skin-derived MCs differ by age, sex and skin of origin of the donors, they share many features, and variability seems to be more strongly influenced by individuals with their selective (epi-)genomes than by the skin of origin (including sex or age) [78]. Although it will have to be experimentally proven, we therefore surmise that CREB plays similar roles in skin MCs in both sexes.

CREB is activated by several kinases, including ERK1/2 [30,79,80,81], and positioned at a hierarchically privileged position based on its ability to drive the expression of a large number of genes. Several of these encode crucial TFs themselves with the ability to regulate plenty of enhancers and promoters on their own. In fact, many immediate early genes (IEGs) are TFs (including members of the AP-1, EGR, and NR4A families). These genes are highly expressed in skin MCs and/or robustly induced by different types of stimulation in an ERK1/2- and CREB-dependent manner [29,30,65,82]. Based on large-scale studies and bioinformatics predictions, AP-1 alone has several tens of thousands of binding sites, for instance, regulating a broad spectrum of physiological and pathological processes, even though only a fraction will be functional in any given cell [83,84,85,86,87,88,89]. Other key TFs that do not belong to the IEG category are also regulated by CREB, including MITF in melanocytes [90,91,92,93,94], in which CREB also becomes phosphorylated upon KIT activation [95,96]. In addition to melanocytes, MITF is also a master regulator of the MC lineage [97,98]. Since MITF transcription in MCs uses a different promoter than in melanocytes [32,33], it remains to be seen whether CREB can regulate MITF abundance in MCs as well. Our preliminary data upon CREB inhibition do not support a strong regulatory effect in MCs, however. Collectively, as a master switch CREB can regulate various genes during homeostasis and activation either directly (by activating transcription from their promoters/enhancers) or indirectly (by supporting the expression of subordinate TFs).

Our study also allows us to further estimate the degree of correspondence between mRNA and protein abundance of the crucial MC receptors studied herein. Overall, the predictive power of mRNA for protein levels has been estimated to be 40% on average but with substantial variance among genes [99].

For KIT, mRNA and protein have been shown to be uncoupled, likely owing to the various levels at which regulation occurs (e.g., translational efficiency, internalization and protein stability) [30,100].

In case of FcεRI, we know from population-based studies that mRNA levels moderately predict cell surface appearance of the α*β*γ_2_ receptor complex [101,102,103], while FcεRI*β* and FcεRIγ do not [75]. Moreover, despite the rather modest correlation based on MCs from many individuals, FcεRI surface density and FcεRIα-specific transcript typically trend in the same direction after microenvironmental changes [50,52,53]. This is reproduced here, as CREB-RNAi led to consistent reduction of FcεRIα protein and transcript. Binding sites for the activating transcription factor (ATF) (which resemble those of CREB [76]) were detected in the FCER1A gene [https://www.genecards.org/cgi-bin/carddisp.pl?gene=FCER1A, last accessed 18 August 2024]. We saw consistent downregulation of its transcript following manipulation of CREB by CREBi or CREB-RNAi. Therefore, it appears possible that CREB binds to these sites and positively regulates the FCER1A gene directly, but clarification will require future efforts.

For MRGPRX2, transcript and protein abundance robustly correlate, and MRGPRX2 expression also gives a good estimate of the receptor’s functional outputs, based on previous studies [49,52,64]. Overall, though the association is not absolute, variability in MRGPRX2 expression better predicts the strength of MRGPRX2 function than variability in FcεRI abundance predicts FcεRI elicited outcomes.

It therefore came as a surprise that, while MRGPRX2 increased following CREB-RNAi, MRGPRX2 elicited exocytosis was potently reduced by the same strategy. To our knowledge, this is the first report that shows this type of uncoupling, identifying CREB as differentially regulating MRGPRX2 at the expressional and functional levels.

Since CREB plays a tremendous role in skin MCs [30,45], it may be envisaged to use CREB inhibitors at low concentrations in combination with other therapies aiming to reduce MC hyperactivity in inflammatory disorders. Combining low concentrations of drugs targeting distinct pathways is a strategy to spare the toxicity of each individual substance and increase overall efficiency [104,105]. It is of interest that 666-15. i.e., the inhibitor used in our study, was in general well-tolerated and effective in vivo [56,57,106,107,108]; it has also been considered for cancer therapy [109]. Exploring the activity of this or related compounds against allergic disorders seems to be a plausible strategy for the future. Another possibility would be to manipulate CREB-regulated targets rather than CREB itself [110]. Considering the potency and versatility of CREB in skin MCs, further knowledge in this area may not only open new therapeutic considerations in mastocytosis or MC leukemia, but also in the context of inflammatory skin disorders. Thus, a detailed understanding the CREB-regulated processes in MCs could advance the treatment of multiple conditions brought about or assisted by MCs.

## 5. Conclusions

Our study finds that the ancient TF CREB is critically involved in the regulation of MC exocytosis through a generic pathway that does not primarily depend on the receptor, which elicits degranulation. In fact, the expression of MRGPRX2 and FcεRI as two major degranulation-competent receptors of skin MCs is modestly regulated by CREB, and trends in opposite directions, while degranulation itself requires CREB in a consistent fashion.

Given the cell type-specific nature of CREB-activated programs [31,35] combined with robust expression of CREB system components in skin MCs, investigations in these cells have the potential to unveil novel aspects of this extensively studied TF.

## Figures and Tables

**Table 1:** Primer pairs used for RT-PCR.

Gene	Forward 5′-3′	Reverse 5′-3′
*CREB1*	GAGAAGCGGAGTGTTGGTGA	TCCGTCACTGCTTTCGTTCA
*KIT*	ACTGTGGCCGTTATCTGGAA	GAAGTGCCCCTGAAGTACCT
*FCERIA*	ACCTGCTGCTGAGTTGAGAT	AAGTGTGGCAGCTGGACTAT
*MRGPRX2*	CGGCCTGGGGAACAGAAAGT	GGATCAGGAAGACCGGGATCA
*HPRT*	GCCTCCCATCTCCTTCATCA	CCTGGCGTCGTGATTAGTGA
*PPIB* *	AAGATGTCCCTGTGCCCTAC	ATGGCAAGCATGTGGTGTTT
*GAPDH*	ATCTCGCTCCTGGAAGATGG	AGGTCGGAGTCAACGGATTT

* The PPIB gene encodes Cyclophilin B.

## Data Availability

No datasets were generated or analyzed during this study.

## Supplementary Materials

The following supporting information can be downloaded at: https://www.mdpi.com/article/10.3390/cells13201681/s1, Figure S1: MCs from male and female individuals show comparable expression of CREB. Figure S2: Pharmacological inhibition of CREB does not affect CREB expression whereas RNAi leads to effective CREB knockdown. Figure S3: CREB is required for skin MC degranulation, as evidenced by CD107a exteriorization.
