# Supplementary material for: CREB Is Critically Implicated in Skin Mast Cell Degranulation Elicited via FcεRI and MRGPRX2
Source: Cells. 2024 Oct 11;13(20):1681. doi: 10.3390/cells13201681 (PMC11506305; doi:10.3390/cells13201681)
Supplement: Supplementary file 1 [file cells-13-01681-s001.zip › cells-3118396-supplementary.pdf]

CREB is critically implicated in skin mast cell degranulation elicited via FcεRI and MRGPRX2

Zhuoran Li [1,2,#], Jean Schneikert [1,2,#], Shiva Raj Tripathi [1,2], Manqiu Jin [1,2], Gürkan Bal [1,2], Torsten Zuberbier [1,2], Magda Babina [1,2,*]

1 Fraunhofer Institute for Translational Medicine and Pharmacology ITMP, Immunology and Allergology IA, 12203 Berlin, Germany;
2 Institute of Allergology, Charité – Universitätsmedizin Berlin, corporate member of Freie Universität Berlin and Humboldt Universität zu Berlin, Hindenburgdamm 30, 12203 Berlin, Germany;
zhuoran.li@charite.de (Z.L.); jean.schneikert@charite.de (J.S.); manqiu.jin@charite.de (M.J.), guerkan.bal@charite.de (G.B.); shiva-raj.tripathi@charite.de (S.T.); torsten.zuberbier@charite.de (T.Z.); magda.babina@charite.de (M.B.)
* Correspondence: magda.babina@charite.de

[#] contributed equally

Supplementary Material

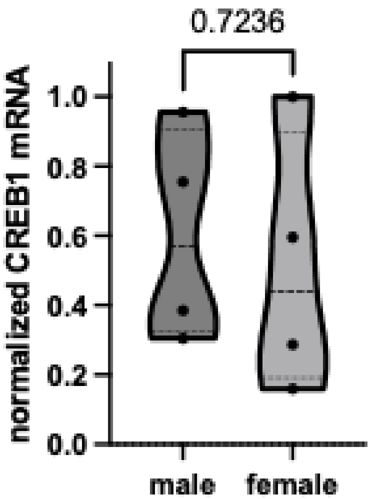

Figure S1. MCs from male and female individuals show comparable expression of CREB. Human MCs were isolated from male foreskin and female breast skin tissue. CREB1 expression was determined by RT-qPCR and normalized against three housekeeping genes as described in Methods. Each dot corresponds to an individual skin MC preparation (donor in the case of breast skin). The data are depicted as violin plots with the p-value given above.

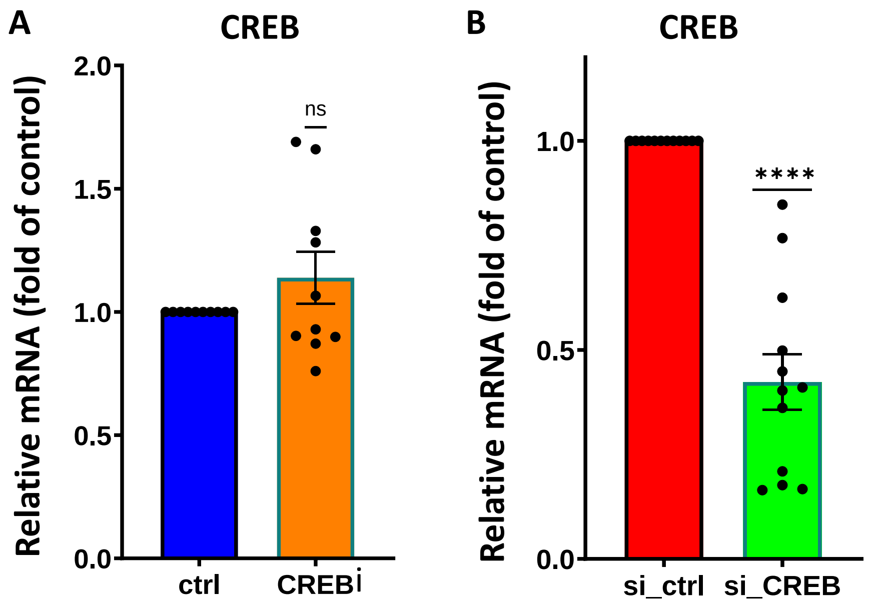

Figure S2. Pharmacological inhibition of CREB does not affect CREB expression whereas RNAi leads to effective CREB knockdown. skin MCs were either pretreated with the CREB inhibitor 666-15 (CREBi) or vehicle (ctrl) (**A**) or transfected with control siRNA (si_ctrl) or a CREB-targeting siRNA (si_CREB) (**B**). Cells were harvested after 2 d and RT-qPCR was performed. Results (normalized to housekeeping genes as described in Methods) are expressed relative to the control set to 1. Results are given as mean ± SEM and individual dots. ****, $p < 0.0001$ using the one sample t-test (A) and Wilcoxon test (B). ns, not significant.

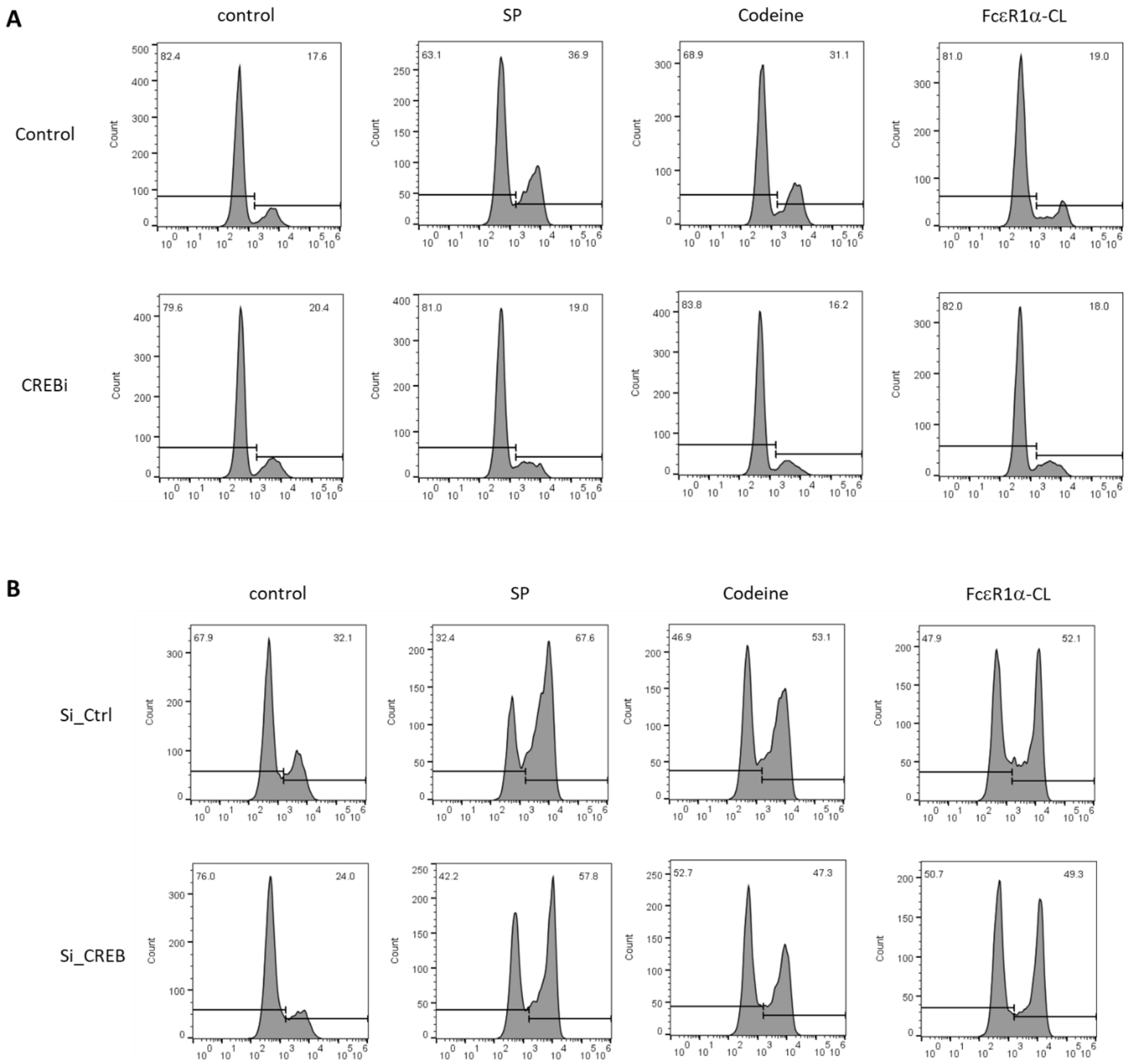

Figure S3. CREB is required for skin MC degranulation, as evidenced by CD107a exteriorization. skin MCs were either pretreated with the CREB inhibitor 666-15 (CREBi) or vehicle (ctrl) (**A**) or transfected with control siRNA (si_ctrl) or a CREB-targeting siRNA (si_CREB) (**B**). Cells were stimulated on day 2 with the stimuli specified above the histograms, stained for CD107a and analyzed by flow-cytometry, as detailed in Methods. The experiments were performed on two independent MC cultures with comparable results.
